# Maternal slaughter and foetal wastage in Nigerian municipal abattoirs: Prevalence, drivers, economic losses and One Health implications

**DOI:** 10.1016/j.onehlt.2026.101508

**Published:** 2026-07-03

**Authors:** Ugochinyere J. Njoga, Emmanuel O. Njoga, James W. Oguttu

**Affiliations:** a Department of Veterinary Obstetrics and Reproductive Diseases, Faculty of Veterinary Medicine, University of Nigeria, Nsukka 410001, Nigeria; b Department of Agriculture and Animal Health, College of Agriculture and Environmental Sciences, University of South Africa, Florida Science Campus, Roodepoort 1709, Johannesburg, South Africa; c Department of Veterinary Public Health and Preventive Medicine, Faculty of Veterinary Medicine, University of Nigeria, Nsukka 410001, Nigeria

**Keywords:** Abattoir surveillance, Food security, Livestock productivity, Pregnancy detection, Sustainable livestock systems, Zoonotic disease transmission

## Abstract

Indiscriminate maternal slaughter and the resulting foetal wastages are persistent but overlooked threats to livestock productivity, food security, and public health in Africa. Comprehensive data on the drivers and consequences of foetal wastages in Nigerian slaughterhouses remain limited, hampering control efforts. A cross-sectional epidemiological study was conducted for six months in four major slaughterhouses in Enugu State, Nigeria, to determine the pregnancy status of 1745 randomly selected female animals (589 cows, 586 does, 570 sows) slaughtered for meat. Univariable analysis assessed associations between species, age, breed, and season (independent variables) and pregnancy (outcome). Variables with *p* ≤ 0.2 were included in the multivariable models, while descriptive statistics were used to summarize the data. Economic losses were estimated based on unit foetal monetary values and adjusted for 5% post-partum mortality. Prevalence of maternal slaughter was 31.7%, 31.1% and 10.5% in goats, cattle, and pigs, respectively. Hot/dry season was associated with pregnancy in goats (AOR = 1.58; 95% CI: 1.03–2.42; *P* = 0.036). The 907 foetuses recovered were mostly in the first trimester (38.3–52.2%), with 18.7–25.6% in the third trimester. Foetal disposal included open dumping (28%), sale for dog food (32%), sale for human consumption (18%), and feed for farmed fish (22%). Net economic loss over the six months this study lasted was US$23,659.94. Major drivers of maternal slaughter and foetal wastage included ignorance of pregnancy status (96%), economic hardship (38%), and high meat demands during festivities (20%). Maternal slaughter and foetal wastage are largely driven by systemic failure in livestock production and processing governance. Maternal slaughter impacts livestock farming profitability and productivity, genetic conservation, food security, zoonotic disease spread, occupational and environmental health negatively. Mandatory ante-mortem pregnancy screening, stakeholder education and improved abattoir governance are imperative to limit maternal slaughter, foetal wastage and the negative impacts.

## Graphical abstract

**Figure:**
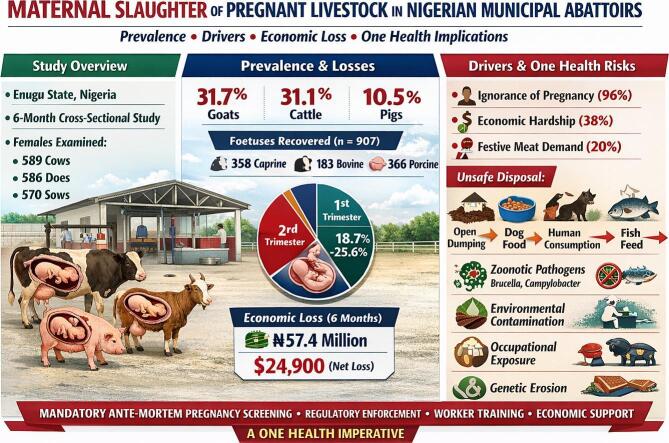

## 1 Introduction

The routine slaughter of reproductive or pregnant female animals remains a persistent yet overlooked challenge in low- and middle-income countries (LMICs), particularly across sub-Saharan Africa. Foetal wastage, defined as the loss of viable foetuses through the slaughter of gravid females [1], [2], compromises livestock productivity by reducing herd replacement, accelerating genetic erosion, and diminishing future meat, milk, and breeding outputs [3]. Consequently, it undermines progress toward the attainment of Sustainable Development Goal 2 (Zero Hunger) by weakening herd expansion, reducing animal protein availability, and threatening long-term food security. Beyond productivity losses, the practice raises serious ethical concerns, exacerbates food insecurity, and imposes substantial but largely hidden economic losses along livestock value chains [4], [5], [6]. Despite these far-reaching consequences, context-specific drivers of foetal wastage remain poorly documented and under-researched in many parts of Africa and LMICs in general. This gap is particularly evident in Enugu State, Nigeria, where limited empirical data constrain evidence-based policy formulation and the implementation of effective intervention measures.

Beyond the immediate production losses, one of the most critical but least emphasized consequences of pregnant animal slaughter is genetic erosion. Each wasted foetus represents the loss of a future breeding animal, accelerating the depletion of already vulnerable livestock genetic resources. In Nigeria, indigenous livestock (cattle, goat, and pig breeds) possess valuable traits such as adaptation to harsh tropical environments and resistance to endemic diseases [7], [8]. Preventable foetal wastage hastens the disappearance of these traits, undermining sustainable livestock production and weakening resilience to climate change adaptations, emerging pathogens, and market instability. This is particularly concerning in the context of global efforts to conserve animal genetic resources as a cornerstone of food security, sustainable animal agriculture and One Health resilience.

In addition to genetic and productivity losses, slaughterhouses represent high-risk interfaces for zoonotic disease transmission, risks that are amplified when gravid animals are slaughtered. Amniotic fluid, placental tissues, and foetal blood may harbour pathogens of veterinary and public health importance., For Nigeria, this issues have been reported for *Brucella* spp. [9], [10], [11], [12], [13], *Campylobacter* spp. [14], [15], [16], [17], [18], [19], [20], pathogenic antibiotic-resistant *Escherichia coli* [21], [22], staphylococci [23], [24] and zoonotic parasites [24], [25], [26], [27], [28], [29], [30]. In many slaughterhouses in Africa, foetal evisceration in gravid carcasses is frequently performed without adequate personal protective equipment, increasing occupational exposure among slaughterhouse workers [6], [31]. Poor disposal of foetal materials further promotes environmental contamination, scavenger attraction, and downstream pathogen transmission risks [6]. In settings with weak regulatory oversight, these practices compromise meat hygiene and contribute to neglected zoonotic and foodborne disease burdens [7], [8], [9].

These public health risks are compounded by food security implications, as foetal wastage translates into compounded losses of animal protein. The premature removal of a gravid dam eliminates current production and future offspring, constraining herd expansion and long-term supply [2]. These losses further widen animal protein deficit in some LMICs, where per capita meat consumption remains among the lowest globally and the average daily protein intake falls below FAO-recommended minimum requirements (≈ 0.6–0.75 g/kg body weight) [31]. Food safety risks are also heightened, as gravid animals are often slaughtered due to poor body condition or reproductive disorders, factors that may compromise carcass quality and increase pathogen contamination. Collectively, foetal wastage undermines both the availability and safety of animal-source foods.

Although multiple studies have estimated foetal wastage across Africa [33], [34], [35], [36], [37], [38], [39], major gaps remain, as most focused narrowly on prevalence while neglecting causative factors, socioeconomic drivers, and broader public health and reproductive implications. In Nigeria, particularly in Enugu State, evidence remains sparse. No published data exist on pig foetal wastage in Enugu State, Nigeria or most parts of the country, despite the growing importance of pig production. The most recent reports on maternal slaughter in goats date back to 2018, when researchers documented 59% prevalence in Nsukka, Enugu State, Nigeria [40]. This study therefore addresses these gaps by determining the prevalence, drivers and economic losses associated with maternal slaughter and foetal wastage at slaughterhouses in Enugu State, Nigeria. The study also elucidates on the factors that predict maternal slaughter among animals slaughtered at selected abattoirs in Nigeria and the implications for livestock production and One Health Science.

## 2 Materials and methods

### 2.1 Study area and design

This cross-sectional epidemiological study was carried out in Enugu State, Southeast Nigeria (6°27′10″N; 7°30′40″E) [41], between January and July 2025. The study spanned six months, encompassing three months of the dry season (January–March 2025) and three months of the rainy season (May–July 2025). The state serves as a major livestock trading and meat-processing hub, receiving animals from multiple regions of Nigeria. Four government-approved slaughter facilities, Ikpa, 9th Mile, Akwata, and New Artisan, were purposively selected because they account for over 80% of legally slaughtered food animals in the state. Each facility operates separate slaughter lines for cattle, goats, and pigs, allowing species-specific monitoring, which reduces misclassification.

### 2.2 Study population and sampling strategy

The study population comprised all female cattle, goats, and pigs presented for slaughter during the study period. Animals slaughtered outside approved facilities were excluded. Systematic random sampling (one in three) was conducted on Wednesdays and Saturdays between 06:00 and 08:00 h, corresponding to peak slaughter periods. The first animal was selected by simple random sampling - a coin toss. This approach minimized selection bias while remaining operationally feasible.

### 2.3 Sample size determination

Minimum sample sizes for the different populations required to detect statistical significance for each species were calculated using the Raosoft® sample size calculator freely available online at http://www.raosoft.com/samplesize.html. A 95% confidence level, 5% margin of error and an estimated annual slaughter population of 200,000 female animals were assumed during the computation. Expected prevalence of 17.4% for cattle [1], 59% for goats [38], and 50% for pigs (due to absence of prior published regional data) were assumed. The calculated minimum sample sizes were 219 for cattle, 365 for goats, and 385 for pigs. However, 589 cows, 586 does, and 570 sows were finally examined to enhance the robustness of the dataset and improve the precision and reliability of the findings.

### 2.4 Data collection

a) Post-mortem pregnancy detection

Following evisceration, reproductive tracts were excised and longitudinally incised for pregnancy assessment [1], [2], [5]. The uterus was examined for presence of foetuses, amniotic fluid, placentation, (placentome in ruminants) or other macroscopic evidence of pregnancy as previously described [2], [5]. Detection of one or more foetuses or foetal tissues constituted definitive evidence of pregnancy and was classified as maternal slaughter. Maternal age was estimated post-mortem using species-specific dentition methods [42], [43], [44].

b) Foetal enumeration and gestational age estimation

Recovered foetuses were carefully enumerated, and sex determined by visual inspection of the external genitalia located at the inguinal region (males) or below the tail base (females). Crown–rump length (CRL) was measured in centimetres using a flexible metric tape from the occipital articulation to the base of the tail for foetal age estimation. Gestational age (days) was estimated using validated species-specific regression equations: X = 2.5 (Y + 21) for cattle [45], X = 2.1 (Y + 17) for goats [46], and X = 3 (Y + 21) for pigs [44], where X represents gestational age and Y represents CRL. Gestational stages were then categorized into trimesters based on species-specific gestation lengths as follows: cattle (∼280 days: first 0–93, second 94–186, third 187–280); goats (∼150 days: first 0–50, second 51–100, third 101–150), and pigs (∼114 days: first 0–38, second 39–76, third 77–114).

c) Questionnaire survey and observational assessment

An 11-item structured closed-ended questionnaire was developed to assess socio-demographic characteristics, professional training status, reasons for slaughtering pregnant animals, and disposal practices for foetuses and gravid uterine contents (Supplementary file *S*1). Content validity was determined as previously reported [30], [47], [48], [49], [50] using a three-member expert panel. Internal consistency reliability check for the instrument of data collection yielded a Cronbach's alpha value of 0.791 (79.1%), which is ≥0.7 (70%) benchmark. After pilot testing among 15 respondents outside the study area, the refined instrument was administered via face-to-face interviews to 50 respondents (butchers, livestock traders, and meat processors) who consented to partake in the study. Foetal disposal practices were corroborated through direct observation within the slaughter facilities.

### 2.5 Data analysis

#### 2.5.1 Statistical analyses

Data collated from completed questionnaires were entered into Microsoft Excel and exported to GraphPad Prism® version 8.0.4 (GraphPad Inc., CA, USA) for statistical analysis. Pregnancy status at slaughter (pregnant = 1; not pregnant = 0) was treated as the binary outcome variable. Descriptive statistics (frequencies and proportions) were used to summarize categorical variables and results presented as tables and figure.

Univariable logistic regression analyses were first conducted separately for each epidemiological variable (age category, breed, and season) for each species (goats, cattle and pigs) as predictors, and pregnancy and maternal slaughter as outcome variables to estimate crude odds ratios (COR) and their 95% confidence intervals (CI). Variables with *p* ≤ 0.2 in univariable analysis were entered into species-specific multivariable logistic regression models. For goats and cattle, age, season and breed were simultaneously included in the multivariable models to adjust for potential confounding. For pigs, all variables were initially entered; however, none demonstrated statistical significance. Reference categories were defined a priori as the youngest age group, wet/rainy season, and minor/other breed category. Adjusted odds ratios (AOR) with their 95% CI were reported.

Confounding was assessed by comparing adjusted odds ratios. A change ≥10% in the AOR after the removal of a suspected confounder was considered indicative of confounding, and such a variable was left in the model. Multicollinearity among independent variables was evaluated using variance inflation factors (VIF), with VIF > 5 considered indicative of problematic collinearity. Variance inflation factors ranged from 1.08 to 1.42 in goats, 1.05–1.36 in cattle, and 1.02–1.31 in pigs, indicating absence of multicollinearity.

Model fit was assessed using the Hosmer–Lemeshow goodness-of-fit test. A non-significant *p*-value (>0.05) was interpreted as adequate calibration. Model diagnostics demonstrated adequate goodness-of-fit across species. For goats, the Hosmer–Lemeshow test yielded χ^2^ = 6.41 (df = 8, *p* = 0.60). For cattle, χ^2^ = 5.87 (df = 8, *p* = 0.66), and for pigs, χ^2^ = 4.92 (df = 8, *p* = 0.77), indicating no evidence of poor model calibration.

The discriminatory ability of the model was further evaluated using the receiver operating characteristic (ROC) curve. The model showed acceptable predictive performance with an area under the curve (AUC) of 0.74 (95% CI: 0.71–0.78).

Prevalence estimates were calculated with exact 95% CI using binomial methods. All statistical tests were two-tailed, and statistical significance was set at *p* < 0.05.

#### 2.5.2 Economic loss estimation and sensitivity analysis

Economic losses associated with foetal wastage were estimated using a deterministic partial budget model based on projected market value of offspring at weaning age. Each recovered foetus was assumed to represent a potential live birth. A base-case 5% post-partum mortality adjustment was applied in accordance with regional survival estimates reported in sub-Saharan Africa.

Species-specific mean market prices were determined through structured market surveys conducted during the study period. Prevailing prices were ₦25,000 per goat kid, ₦80,000 per calf, and ₦40,000 per piglet at early weaning post colostrum ingestion. All economic values were converted using the official exchange rate of ₦1535 to US$1 at the time of analysis. Net economic loss per species was calculated as: Net loss = (Number of foetuses x Unit price) minus 5% mortality adjustment [5], [6], [51]. To account for price volatility and uncertainty in survival assumptions, a one-way sensitivity analysis was performed under three scenarios: a) optimistic scenario: +10% market price, 3% mortality; b) base-case scenario: observed market price, 5% mortality and c) conservative scenario: −10% market price, 10% mortality. This approach enabled estimation of lower and upper bounds of economic impact. The model did not incorporate long-term productivity losses (future reproductive output, milk yield, or genetic gain) and therefore represents conservative short-term financial estimates.

#### 2.5.3 Ethical approval and biosafety

Ethical approval was obtained from the Institutional Animal Care and Use Committee (IACUC) of the Faculty of Veterinary Medicine, University of Nigeria, Nsukka (Ref. No: FVM-UNN-IACUC-2024-12/115). The study complied with national ethical guidelines for animal research and human participation. Informed consent was obtained from all interviewed respondents. Participation was voluntary, and anonymity and confidentiality were maintained throughout.

All personnel involved in the study adhered to abattoir biosecurity regulations. Personal protective equipment (PPE), including coveralls, gloves, waterproof boots, masks, and goggles, were worn throughout sampling. Disinfection of tools and work surfaces was carried out with 2% sodium hypochlorite after each session, and biological wastes were disposed in accordance with national abattoir hygiene standards.

## 3 Results

### 3.1 Socio-demographic profile of respondents

The socio-demographics of the respondents are summarised and presented in Table 1. Majority of the respondents were males (96%, 48/50). Seventeen (34%, 17/50) had over 10 years working experience while only 4 (8%) had formal training in hygienic carcass processing (Table 1). All the 50 respondents had slaughtered pregnant animals for meat.

**Table 1:** Socioeconomic characteristic of respondents (*n* = 50) surveyed for slaughter of pregnant goats or pigs in Nsukka, Nigeria.

Socioeconomic characteristic	Frequency (%)
*Gender*	
Male	48 (96)
Female	2 (4)
*Age category (years)*	
18–35	19 (38)
36–45	16 (32)
> 45	15 (30)
*Job description*	
Butchers	11 (22)
Carcass processors	19 (38)
Livestock traders	20 (40)
*Marital status*	
Single	16 (32)
Married	34 (68)
*Working experience (years)*	
< 5	11 (22)
5–10	22 (44)
> 10	17 (34)
*Highest educational level attained*	
No formal education	10 (20)
Primary	2 (4)
Secondary	22 (44)
Tertiary	16 (32)
*Have had formal training*	
Yes	4 (8)
No	46 (92)

### 3.2 Drivers of maternal slaughter and foetal wastage

**Fig. 1:**
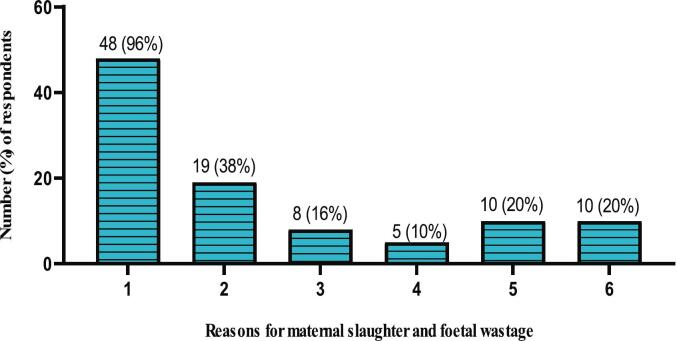
Illustration of the major reasons for slaughtering or selling pregnant livestock for meat at slaughterhouses in Enugu State, Nigeria.

According to the respondents, the major reasons for slaughtering gravid animals for meat included ignorance of the pregnancy status (96%), economic hardship (38%) and increased demand for meat during festive periods (20%). Additional reasons for maternal slaughter are presented in Fig. 1.

1 = Ignorance of the pregnancy status, 2 = Economic hardship, 3 = Dystocia cases, 4 = Buyers' preference for large-sized animals. 5 = Emergency slaughter due to injuries or diseases, 6 = Increased meat demand during festive periods.

Disposal of eviscerated foetuses and uterine contents.

Following evisceration, foetal materials were disposed of through several channels. Open refuse dumping accounted for 28% (14/50) of disposal methods employed by the respondents. A slightly higher proportion (32%, 16/50) of respondents disposed of foetal material by selling them to be used in dog food preparation. The rest of the respondents sold foetal material to be used to prepare a local delicacy commonly referred to as meat pepper soup (18%, 9/50) or as feed for farmed fish (22%, 11/50).

### 3.3 Prevalence of maternal slaughter

A total of 1191 goats, consisting of 586 (49.20%) does and 605 (50.80%) bucks, were slaughtered during the 6 months study period. Similarly, 1185 pigs consisting of 570 (48.10%) sows and 615 (51.90%) boars were slaughtered over the study period. For cattle, 1421 animals comprising of 589 (41.31%) cows and 834 (58.69%) bulls were slaughtered.

Out of the total number of females slaughtered, 186 (31.7%) does, 183 (31.1%) cows and heifers, and 60 (10.5%) sows were pregnant on post-mortem examination. There was a significant association (*p* < 0.05) between the species and the number of pregnant animals slaughtered, with goats (31.7%, 186/586) and cattle (31.1%, 183/589) having the highest proportion of pregnant animals slaughtered. Pigs had the lowest proportion of pregnant females that were slaughtered (10.5%, 60/570) (Table 2).

**Table 2:** Overall prevalence of pregnant animals slaughtered for meat at municipal slaughterhouses in Enugu State, Nigeria.

Species	Females slaughtered (n)	Pregnant females (n)	Prevalence	χ^2^-value	P-value
Goat	586	186	31.7	93	< 0.001⁎
Pig	570	60	10.5		
Cattle	589	183	31.1		

⁎ = Statistical significant p-value, Chi-square statistics.

The distributions of pregnant female animals slaughtered by some epidemiological factors are presented in Table 3. In ruminants (goats and cattle), there were significant statistical associations (*p* < 0.05) between age and season, and the number of maternal slaughters (Table 3).

**Table 3:** Distribution of maternal slaughters (pregnant female animals slaughtered) in Enugu State, Nigeria, by various epidemiological factors.

Epidemiological factors	Epidemiological variables	Females sampled (n)	Females pregnant (n)	Prevalence	χ-value	P-value
GOAT						
Age	≤ 2 years	92	21	22.8	6	0.049⁎
	2–4 years	405	142	35.1		
	> 4 years	89	23	25.8		
Breed	Red Sokoto	386	133	34.5	6.1	0.047⁎
	Kano brown	156	49	31.4		
	Other breeds	37	7	18.9		
Season	Wet/rainy	168	40	23.8	6.8	0.009⁎
	Hot/dry	418	146	34.9		
CATTLE						
Age	≤ 4 years	125	38	30.4	7.1	0.029⁎
	4–8 years	218	81	37.2		
	> 8 years	246	64	26		
Breed	White Fulani	441	143	32.4	1.6	0.665
	Sokoto Gudali	68	18	26.4		
	Red Bororo	42	12	28.6		
	Other breeds	38	10	26.3		
Season	Wet/rainy	241	64	26.6	2.9	0.048⁎
	Hot/dry	348	119	34.2		
PIGS						
Age	≤ 2 years	112	12	10.7	0.88	0.956
	2–4 years	276	28	10.1		
	> 4 years	182	20	10.9		
Season	Wet/rainy	277	27	9.7	0.35	0.556
	Hot/dry	293	33	11.3		

⁎ = Statistical significant *p*-value; Chi square test (GraphPad Prism®, version 8.0.4, CA, USA).

### 3.4 Multivariable logistic regression analysis

Variables with *p* ≤ 0.20 in univariable analysis were included in species-specific multivariable models. After adjustment for age, breed, and season, only season was significantly associated with pregnancy in goats, with goats slaughtered in the hot/dry season almost twice as likely to be pregnant at slaughter (AOR = 1.58; 95% CI: 1.03–2.42; *P* = 0.036). In cattle and pigs, none of the independent predictors were significantly associated with pregnancy (Table 4).

**Table 4:** Results of the multivariable logistic regression analysis to determine the predictors of pregnancy status at slaughter in goats, cattle, and pigs in Enugu State, Nigeria.

Variable	Category	AOR	95% CI	P-value
GOAT				
Age	<2 years (Ref.)	1(Ref.)	–	–
	2–4 years	1.61	0.93–2.79	0.089
	>4 years	1.05	0.51–2.15	0.89
Breed	Other	1(Ref.)	–	–
	Red Sokoto	2.02	0.84–4.83	0.11
	Kano Brown	1.82	0.72–4.59	0.21
Season	Wet	1(Ref.)	–	–
	Hot/Dry	1.58	1.03–2.42	0.036⁎
CATTLE				
Age	< 4 years (Ref.)	1.00	–	–
	4–8 years	1.18	0.70–1.99	0.53
	>8 years	0.75	0.43–1.30	0.30
Breed	Other	1 (Ref.)	–	–
	White Fulani	1.28	0.57–2.86	0.55
	Sokoto Gudali	0.98	0.35–2.71	0.97
	Red Bororo	1.09	0.36–3.25	0.88
Season	Wet	1(Ref.)	–	–
	Hot/Dry	1.36	0.94–1.96	0.10
PIGS				
Age	<2 years	1(Ref.)	–	–
	2–4 years	0.90	0.43–1.89	0.78
	>4 years	0.99	0.45–2.14	0.97
Season	Wet	1(Ref.)	–	–
	Hot/Dry	1.13	0.64–1.98	0.67

⁎ = Statistical significant p-value, AOR = Adjusted Odds Ratio, CI = Confidence Interval.

### 3.5 Prevalence of foetal wastages

Of the 186 pregnant goats slaughtered, 34 (18.3%), 132 (70.9%) and 20 (10.8%) had single, twin and triplet pregnancies, respectively. Consequently, a total of 358 caprine foetuses were recovered from the 186 pregnant goats slaughtered. Similarly, 366 porcine foetuses and 183 bovine foetuses were recovered from the 60 sows and 183 cows slaughtered, respectively. On the foetal sex, 53.1% (190/358), 55.7% (102/183) and 27.9% (212/366) of the foetuses were females for goats, cattle and pigs respectively. Overall, 907 caprine, bovine and porcine foetuses were wasted within the 6 months study. The distributions of the foetal ages across the three species are presented in Table 5 while the images of various species of foetuses recovered at various gestational ages are shown in supplementary file *S*2.

**Table 5:** The distributions of the foetuses by trimester/gestational age across the animal species surveyed.

Species	Foetuses recovered (n)	Gestational ages of the foetuses recovered		
		First trimester (%)	Second trimester (%)	Third trimester (%)
Goat	358	187 (52.2)	104 (29.1)	67 (18.7)
Cattle	183	91 (49.7)	54 (29.5)	38 (20.8)
Pigs	366	140 (38.3)	132 (36.1)	94 (25.6)

### 3.6 Economic loss estimates

The total aggregate economic loss from foetal wastage across goats, cattle, and pigs was ₦38,230,000 (US$24,905.54)**.** Goats accounted for ₦8,950,000 (US$5830.62)**,** while cattle and pigs each accounted for ₦14,640,000 (US$9537.46)**.** Following a 5% possible post-partum mortality adjustment, the total deduction amounted to ₦1,911,500 (US$1245.60)**.** The resulting net economic loss across all species was therefore ₦36,318,500 (US$23,659.94)**.** After adjustment, the species-specific losses were ₦8,502,500 (US$5538.78) for goats, and ₦13,908,000 (US$9060.59) each for cattle and pigs (Table 6).

**Table 6:** Economic loss estimation associated with caprine, bovine and porcine foetal wastages at municipal slaughter facilities in Enugu State, Nigeria.

Species	Number of Foetuses	Unit Cost	Aggregated Costs	Minus 5% Post-Partum Mortality	Actual Cost per Species (After 5% Deduction)
Goat	358	₦25,000 (US$16.29)	₦8,950,000 (US$5830.62)	₦447,500 (US$291.53)	₦8,502,500 (US$5538.78)
Cattle	183	₦80,000 (US$52.12)	₦14,640,000 (US**$**9537.46)	₦732,000 (US$476.87)	₦13,908,000 (US$9060.59)
Pig	366	₦40,000 (US$26.06)	₦14,640,000 (US$9537.46)	₦732,000 (US$476.87)	₦13,908,000 (US$9060.59)

One US$ = ₦1535.

## 4 Discussion

The study demonstrates high prevalence of maternal slaughter, with 31.7% of does, 31.1% of cows, and 10.5% of sows found pregnant at slaughter. These figures align with, and in some cases exceed, reports from other regions of Nigeria, where caprine and bovine foetal wastage rates ranging between 20% and 45% have been documented [33], [34], [35], [36], [37], [38], [39], [40]. Similar patterns have been reported in sub-Saharan Africa, including Ghana and Uganda where 18.4% [37] and 21.9% [3] prevalence of bovine foetal wastages were reported, respectively. These findings indicate that the problem is systemic across LMICs. However, the inclusion of pigs in the present study addresses an important gap, as porcine foetal wastage has rarely been documented in Nigeria. The findings therefore extend the national evidence base and confirm that maternal slaughter is not species-restricted but rather a cross-cutting livestock systems failure with significant livestock productivity, One Health, and socio-economic ramifications.

From a public health perspective, the slaughter of gravid animals creates a high-risk interface for zoonotic transmission. The recovery of 907 foetuses across species indicates a high exposure of abattoir workers to amniotic fluids, placental tissues, and foetal blood, which are materials known to harbour zoonotic pathogens such as *Brucella* spp., *Campylobacter* spp., pathogenic *Escherichia coli*, and *Staphylococcus aureus* [21], [22], [23], [24], [25], [26], [27], [28], [29], [30]. The zoonotic risks are further compounded in this study, as 92% of respondents lacked formal training in hygienic carcass processing. Additionally, unsafe foetal disposal practices, including open refuse dumping and sale for dog food or human consumption, amplify environmental contamination and foodborne transmission risks. Comparable studies in Nigeria and East Africa have similarly highlighted inadequate biosecurity and poor waste management as drivers of brucellosis and campylobacteriosis spread [6], [35], [36], [37], [38], [39], [40], [41]. Thus, maternal slaughter is not merely an animal production issue but a One Health concern that perpetuates occupational hazards, environmental dissemination of pathogens, and unsafe food production and processing systems. Strengthening ante-mortem inspection, enforcing hygienic disposal protocols, and mandating structured abattoir worker training are immediate practical interventions.

The implications of foetal wastages found in this study for livestock productivity and food security are profound. A total of 907 potential replacement animals were lost within six months, translating into a net economic loss of ₦36.32 million (US$23,659.94). This monetary estimate likely underrepresents the true long-term production loss, as it excludes future reproductive potential, milk yield, genetic gain, and multiplier effects along the value chain. Nigeria already faces a substantial animal protein deficit, with per capita consumption below global averages and recommended dietary protein thresholds [33]. The removal of gravid dams may simultaneously diminish present meat yield and future herd expansion capacity, thereby compounding protein scarcity and undermining national food security strategies. Earlier studies conducted in other countries have demonstrated that foetal wastage contributes to stagnation of national herd growth and exacerbates reliance on imports [3], [37], [52], [53]. Therefore, preventing maternal slaughter through compulsory ante-mortem pregnancy screening of all female animals intended for slaughter could be a cost-effective livestock development strategy that aligns with Sustainable Development Goals 2 toward zero hunger and poverty reduction for all by 2030.

These findings also align with global livestock sustainability and regulatory frameworks. Reducing maternal slaughter contributes directly to the objectives of Sustainable Development Goal 2, which seek to end hunger and strengthen sustainable food production systems through improved livestock productivity and resilience [54]. International welfare standards issued by the World Organization for Animal Health emphasize that animals intended for slaughter must undergo proper ante-mortem inspection and that animals in advanced pregnancy should not be slaughtered except for justified veterinary reasons [54], [55]. Similarly, sustainability frameworks promoted by the Food and Agriculture Organization of the United Nations highlight the importance of protecting breeding stock, reducing avoidable reproductive losses, and strengthening livestock value-chain governance to safeguard long-term food security and livestock productivity [32]. Aligning slaughterhouse management practices in Nigeria with these international guidelines could therefore reduce foetal wastage while supporting national livestock development and public health protection.

Genetic erosion represents another critical but often overlooked consequence maternal slaughter and its consequent foetal wastage. The high proportion of female foetuses recovered, 53.1% in goats and 55.7% in cattle, indicates a substantial loss of future breeding stock. Indigenous breeds such as Red Sokoto goats and White Fulani cattle possess adaptive traits including heat tolerance, disease resistance, and capacity to thrive under low-input systems [7], [8]. Persistent removal of gravid females accelerates depletion of these genetic resources, narrowing the gene pool and reducing resilience to climate variability and emerging pathogens. Comparable concerns have been raised in FAO-led assessments of animal genetic resources globally [32]. The observed higher prevalence during the hot/dry season further suggests market-driven destocking under feed scarcity conditions, which may disproportionately affect productive females. Practical solutions include community-based breeding programs, incentives for retaining pregnant animals such as provision of animal feed, and integration of pregnancy diagnosis into routine livestock market screening.

The prominence of economic hardship as one of the socioeconomic drivers of maternal slaughter and foetal wastages underscores structural vulnerabilities in livestock value chains. In many LMIC contexts, farmers liquidate pregnant stock to meet urgent financial needs due to lack of credit access or insurance mechanisms. Instituting livestock microcredit schemes, emergency veterinary obstetric services, and subsidized pregnancy diagnosis (e.g., portable ultrasonography or rectal palpation by trained personnel) could significantly reduce inadvertent slaughter. Enforcement of existing meat inspection regulations should be strengthened to prohibit slaughter of advanced pregnancies except on veterinary grounds. Additionally, public awareness campaigns targeting traders and butchers can reframe gravid animals as a national high-value breeding assets rather than merely an immediate meat commodities.

Finally, the trimester distribution data, showing that 18.7% of goat, 20.8% of cattle, and 25.6% of pig foetuses were in the third trimester, raise serious ethical and welfare concerns. Advanced gestational slaughter intensifies public perception issues regarding animal welfare and may contravene international humane slaughter guidelines. Countries such as the United Kingdom and members of the European Union enforce stricter oversight on slaughter of late-term pregnancies [54], offering regulatory models adaptable to the Nigerian context. Integrating digital traceability systems, routine pregnancy screening at lairage, and sanctions for non-compliance would modernize abattoir governance.

### 4.1 Limitations of the study

This study was limited to four government-approved slaughter facilities in one state in Nigeria, which did not account for maternal slaughter and foetal wastage in informal and clandestine slaughter settings. This may underestimate the true magnitude of the problem and limits generalizability. In addition, pregnancy detection relied solely on post-mortem macroscopic examination, which may have missed very early gestations, potentially leading to further underestimation. Notwithstanding these limitations, the study provides valuable information that could guide policy formulation to limit maternal slaughter, foetal wastages and the untoward economic and One Health implications in Nigeria and other LMICs.

## 5 Conclusion

Maternal slaughter and foetal wastage in the study area are multidimensional threats spanning zoonotic disease risk, food (protein) insecurity, economic loss, genetic erosion, and ethical governance. Maternal slaughter and the resulting foetal wastages are driven primarily by undiagnosed pregnancies and structural economic vulnerabilities within livestock value chains, reflecting systemic gaps in surveillance, regulation, and producer support. Addressing this challenge demands mandatory ante-mortem pregnancy screening, strengthened regulatory enforcement, and structured certification and biosecurity training for abattoir personnel to curtail preventable foetal losses and occupational health hazards. Complementary economic interventions, including livestock credit, insurance schemes, and emergency veterinary support, are essential to mitigate distress-driven sales of gravid animals. Embedding these integrated measures within national livestock transformation and food security frameworks under a coordinated One Health approach is imperative to safeguard genetic resources, enhance sustainable livestock productivity, and protect public health.

## CRediT authorship contribution statement

**Ugochinyere J. Njoga:** Writing – review & editing, Resources, Methodology, Investigation, Data curation. **Emmanuel O. Njoga:** Writing – review & editing, Writing – original draft, Supervision, Resources, Methodology, Investigation, Formal analysis, Data curation, Conceptualization. **James W. Oguttu:** Writing – review & editing, Supervision, Resources, Project administration, Formal analysis.

## Ethics statement

The Institutional Animal Care and Use Committee (IACUC) of the Faculty of Veterinary Medicine, University of Nigeria, Nsukka (Ref. No: FVM-UNN-IACUC-2024-12/115) granted ethical approval for this work on 09 December 2024. The study adhered to the revised Declaration of Helsinki (World Medical Association, 2013). Informed consent was obtained from all interviewed participants.

## Funding

This research did not receive any specific grant from funding agencies in the public, commercial, or not-for-profit sectors.

## Declaration of competing interest

All the authors have no conflict of interest to declare.

## Data Availability

The data that support the findings of this study are contained within the paper and its supplementary file.

## Appendix A Supplementary data

Supplementary material 1: Copy of the questionnaire used during the study

Supplementary material 2: Animal foetuses at various stages of gestation recovered for slaughterhouses in Enugu State, Nigeria
