# Supplementary material for: Maternal slaughter and foetal wastage in Nigerian municipal abattoirs: Prevalence, drivers, economic losses and One Health implications
Source: One Health. 2026 Jul 3;23:101508. doi: 10.1016/j.onehlt.2026.101508 (PMC13356649; doi:10.1016/j.onehlt.2026.101508)
Supplement: Supplementary file 1 — Supplementary material 1: Copy of the questionnaire used during the study [file mmc1.docx]

**UNIVERSITY OF NIGERIA, NSUKKA**

**FACULTY OF VETERINARY MEDICINE**

**DEPARTMENT OF VETERINARY PUBLIC HEALTH AND PREVENTIVE MEDICINE**

**Questionnaire survey on slaughter of pregnant animals for meat among slaughterhouse workers in Enugu State, Southeast, Nigeria**

Please, tick the appropriate option(s) provided against each of the questions or information below

| **S/N** | **Questions or information required** | **Response** |
| --- | --- | --- |
| **1.** | **Gender** |  |
|  | Male |  |
|  | Female |  |
| **2.** | **Job description*** |  |
|  | Butchers |  |
|  | Carcass processors |  |
|  | Livestock traders |  |
| **3.** | **Working experience (years)** |  |
|  | Less than 5 |  |
|  | 6-10 |  |
|  | Above 10 |  |
| **4.** | **Have had training on modern carcass/meat processing** |  |
|  | Yes |  |
|  | No |  |
| **5.** | **Highest educational level attained** |  |
|  | No formal education |  |
|  | Primary education |  |
|  | Secondary education |  |
|  | Tertiary education |  |
| **6.** | **Reasons for sale or slaughter of pregnant cows for meat*** |  |
|  | Disease condition |  |
|  | Emergency slaughter |  |
|  | Feed scarcity |  |
|  | Economic hardship |  |
|  | Buyers preference for large-sized animals |  |
|  | Ignorance of the pregnancy status of the animal |  |
|  | Others (Please, specify) |  |
| **7.** | **Method of disposal of eviscerated foetuses*** |  |
|  | Open refuse dump |  |
|  | Sold for preparation of dog food |  |
|  | Fed to pigs/cultured fished |  |
|  | Sold for human consumption |  |
|  | Disposed by incineration |  |
|  | Disposed by burial |  |
|  | Others (Please specify) |  |

*Respondents can advance more than one response
