# Supplementary material for: Maternal slaughter and foetal wastage in Nigerian municipal abattoirs: Prevalence, drivers, economic losses and One Health implications
Source: One Health. 2026 Jul 3;23:101508. doi: 10.1016/j.onehlt.2026.101508 (PMC13356649; doi:10.1016/j.onehlt.2026.101508)
Supplement: Supplementary file 2 — Supplementary material 2: Animal foetuses at various stages of gestation recovered for slaughterhouses in Enugu State, Nigeria [file mmc2.docx]

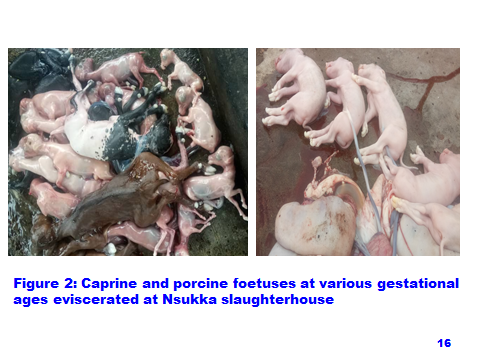

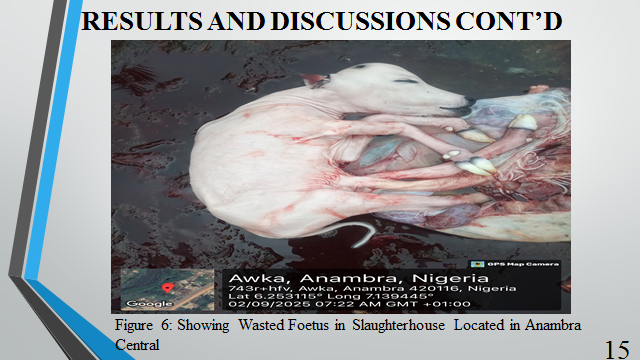

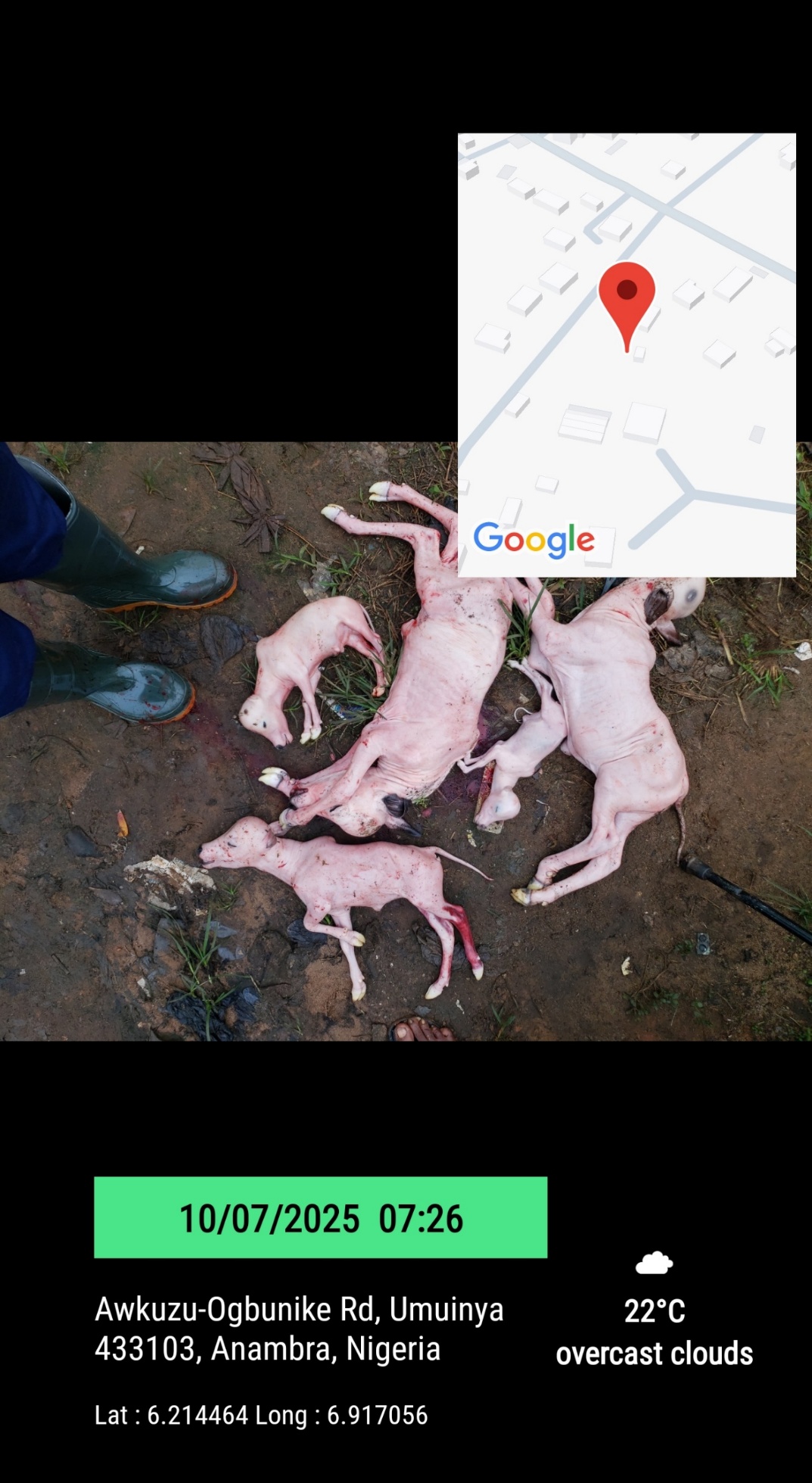


Supplementary file (*S2)*: Bovine, caprine and porcine foetuses at various stages of gestation recovered for slaughterhouses in Enugu State, Nigeria
